# Supporting Breastmilk Feeding for Infants in Foster Care: A Scoping Review

**DOI:** 10.1111/mcn.13810

**Published:** 2025-02-10

**Authors:** Vicky Mitchell, Marianne White, Shona Shinwell, Camila Biazus‐Dalcin

**Affiliations:** ^1^ Mother, Infant and Child Health Research Group University of Dundee Dundee Scotland; ^2^ Infant Feeding Advisor NHS Tayside Dundee Scotland

**Keywords:** breastfeeding, breastmilk, health care professionals, infant, policies, social care professionals, substance use

## Abstract

Worldwide, around 2.7 million children are not in the care of their parents, and access to breastmilk is often absent from foster care policies. We aimed to explore the evidence available on how foster families, health and social workers and mothers with infants in care can be supported in providing breastfeeding and expressed breastmilk (EBM), and to identify barriers and facilitators for breastfeeding and EMB in foster care. The JBI methodology for scoping reviews was used. Three academic databases and grey literature were searched in March 2023, and data extraction charts were used. The findings were synthesised using thematic analysis. In total, 11 papers were included, 5 peer‐reviewed papers and 6 from the grey literature. Five themes were identified in the analysis: ‘Is this safe?’, ‘Substance use: Protecting the breastfeeding rights of mothers and infants’, ‘Making milk accessible through breastfeeding and EBM’, ‘Where are the policies’? and ‘Attitudes around breastfeeding’. The findings showed concern from foster parents around the safety of breastmilk and the challenges of supporting breastmilk provision when infants are in foster care. Training, positive attitudes and multi‐disciplinary team involvement can support breastfeeding and the breastfeeding rights for infants in foster care. Health and social care professionals who support mothers and foster families with breastfeeding and EBM feeding lack knowledge and guidance in how to do this safely and with a rights‐based approach. We found that facilitating breastfeeding is not prioritised when an infant is placed into foster care and that the breastfeeding rights of mothers and infants require urgent attention in policies and guidelines to facilitate safe and person‐centred infant feeding.

## Summary

- Foster carers need further support to enhance their knowledge regarding the safety of handling and giving expressed breastmilk.
- Mothers who wish to continue breastfeeding when their infant is placed in foster care need support from services, if no other risk factors exist.
- Multi‐disciplinary collaboration across health and social care is required to support provision of breastmilk to infants in foster care.
- Evidence‐based guidelines are necessary to facilitate mothers with their establishment and continuation of breastfeeding and expressed breastmilk for their infants in foster care.

## 1 Introduction

Worldwide, it is estimated that 2.7 million children are not in the care of their parents (Petrowski, Cappa, and Gross 2017). However, quantifying the number of children living in out‐of‐home care settings, such as foster care, is challenging, and others estimate the figure to be closer to 5.37 million (Desmond et al. 2020). Foster care is a care arrangement, which can be short or long‐term, in which a child lives with a foster family until their ongoing care plan is decided or children may remain in placements until adulthood (UK Fostering 2022). Infants are removed from the care of their parents through a legal care order and this happens when health and social care professionals agree that under their parent's care, the infant has either suffered or is at risk of harm and/or neglect. Alternatively, parents may voluntarily choose to place their infant into the care of welfare services so they may have time to receive additional support and intervention to improve their parenting abilities (NSPCC 2024).

In the UK, the number of infants living with foster families is on the rise (Raab, McGhee, and Macintyre 2020), with children aged four and under comprising the largest proportion of child protection orders Bunting et al. 2017). Internationally, similar trends have been observed (Högberg et al. 2019; Marsh et al. 2017; O'donnell et al. 2016). Mothers who lose custody of their infant report experiencing significant long‐term negative emotions and grieving the loss of their children. (Everitt, Fenwick, and Homer 2015; Blythe et al. 2021). Children who are brought up in the care system often have poorer outcomes leading to a negative life trajectory compared to their peers who did not grow up in foster care (Gypen et al. 2017).

The World Health Organisation (WHO) recommends that breastfeeding be initiated within the first hour of birth, exclusively for the first 6 months of life, and continued until 2 years of age, or longer (World Health Organization 2022 Breastmilk consumption, including breastfeeding and access to breastmilk, is important in supporting child health, including protecting against sudden infant death syndrome (Vennemann et al. 2009), childhood cancers (Su et al. 2021), and Type 2 diabetes (Horta, Loret de Mola, and Victora 2015). Breastfeeding plays a role in an infant's immunity by regulating the integrity of their gut barrier and training their developing immune system (Dawod, Marshall, and Azad 2021). However, infants who enter foster care are less likely to be breastfed or have access to breastmilk (Köhler et al. 2015; Hunt et al. 2022), which is likely to contribute to poorer health by impacting on their physical, psychological, neurocognitive and nutritional developmental outcomes (Tooley, Makhoul, and Fisher 2016).

Breastfeeding also has long‐term physical and psychological importance for mothers (Victora et al. 2016; Vennemann et al. 2009). Mothers who breastfeed reportedly experience lower rates of postpartum depression and have increased confidence in their ability to mother (Modak, Ronghe, and Gomase 2023). Moreover, a cohort study with 7223 Australian mothers over 15 years showed that breastfeeding may provide a protective effect against maternal child maltreatment, particularly in reducing the risk of child neglect (Strathearn et al. 2009). The physical contact of breastfeeding enables mothers to learn and react to their baby's cues, fostering bonding and emotional regulation development in the infant (Krol and Grossmann 2018; Modak, Ronghe, and Gomase 2023).

The United Nations Convention on the Rights of the Child (UNCRC) protects an infant's right to breastfeeding (United Nations 2022). Despite this, the breastfeeding rights of mothers and infants are often absent from policies that shape foster care (Critchley et al. 2021). In circumstances that prevent a mother from directly breastfeeding, professionals should promote feeding expressed breastmilk (EBM) which is recommended over infant formula (McCloskey and Karandikar 2019). Increasing the understanding of professionals involved in the field of breastfeeding and infant health, as well as in child protection and social work, about the importance of breastfeeding for infants in foster care, would improve support for the breastfeeding rights of women and children (Critchley et al. 2021).

This scoping review aims to explore (1) the evidence available on how health and social care workers, foster families and mothers with infants in care can be supported in providing breastfeeding and EMB and (2) identify barriers and facilitators for breastfeeding and EBM in foster care. A preliminary search of MEDLINE, the Cochrane Database of Systematic Reviews and JBI Evidence Synthesis was conducted, and no current or underway systematic reviews or scoping reviews were identified.

## 2 Methods

### 2.1 Research Design

The scoping review was conducted in accordance with the JBI methodology for scoping reviews (Peters et al. 2020). The registered scoping review protocol is available on Open Science Framework Registries (https://osf.io/mv6k4).

### 2.2 Identifying Literature

The search strategy aimed to locate both peer‐reviewed and grey literature. An initial limited search of MEDLINE and CINAHL was undertaken to identify articles on the topic to use to develop a full search strategy for CINAHL, PUBMED and MEDLINE databases based on their keywords. The same strategy was used to find grey literature in the first five pages of both Google and Google Scholar. The search was conducted in March 2023. Keywords and synonyms of *‘breastfeeding’* and *‘foster care’* were used. Two limiters were applied to the search: (1) papers written in English and (2) papers published before 1994. This date was included to align with the UK's introduction of the UNICEF Baby Friendly Initiative (UNICEF 2016) (Table 1).

**Table 1:** Search terms for electronic database searching.

Breastfeeding *OR* breast‐feeding *OR* breast feeding *OR* breastmilk *OR* breast‐milk *OR* breast milk *OR* complementary feeding *OR* combi feeding *OR* combination feeding *OR* lactation *OR* lactating *OR* expressed breastmilk *OR* expressed breast milk *OR* expressed breast‐milk *OR* EBM
*AND*
Foster care *OR* foster care system *OR* fostering *OR* out‐of‐home care *OR* kinship care
LIMITS
Include only papers publish in English
*AND*
Exclude papers published before 1994

### 2.3 Study Selection

Title and abstracts of all retrieved papers were screened against the inclusion criteria to provide a short list of articles for full‐text review. Two reviewers independently screened all articles at each screening stage to ensure a systematic and rigorous process. A third independent reviewer resolved any disputes between the reviewers. Identified grey literature was reviewed using the same process. The eligibility criteria (Table 2) followed the PCC framework (population, concept and context). Papers were not excluded based on their design. The reference list of all included sources was screened for additional papers.

**Table 2:** Eligibility criteria.

Inclusion	Exclusion
Population: Social workers, foster families or any maternity services worker delivering support, advice, or services for infants being removed into care on the topic of breastfeeding or expressed breastmilk. *OR* Mothers who have had an infant removed between the ages of 0–2 years.	Population: Adoptive parents *OR* Main carer of the infant who plans to breastfeed the child in their care. *OR* Mothers involved in child protection/social services but who have full‐time care of their infants. *OR* Mothers who have an infant removed after the infant's 1st baby.
Concept: Focuses on supporting infants removed from their birth mother's care to be breastfed or EBM.	Concept: Formula milk, or the provision of donor human milk. *OR* Foster mothers' breastfeeding or expressing milk for infants in their care. *OR* Focus on the weaning stage.
Context: Infants being removed into foster care (including kinship care). *OR* Mother breastfeeding in contact visits or expressing breastmilk to be transported to her infant. English language. Papers published since 1994 onwards	Context: Infants who have been adopted. *OR* Non‐English language. *OR* Papers published before 1994.

### 2.4 Charting the Data

Data were charted from all peer‐reviewed and grey literature sources by two reviewers using a data extraction tool. Data were charted according to the type of source: peer‐reviewed literature and grey literature (Tables 3 and 4). The data extracted included specific details about the participants, concept, context, study methods and key findings. Barriers and facilitators of breastfeeding and EBM support for mothers and infants in foster care were also explored (Figure 2). No authors of papers were required to be contacted.

### 2.5 Synthesising the Results

Data analysis used Braun and Clarke (2019) six‐step thematic analysis method. The steps followed were familiarisation of the data which involved VM, MW and SS reading and rereading the full texts of the included papers. The initial coding phase was completed by VM by close reading of each paper and noting key points or similarities of information in relation to the review questions. The initial theme development, reviewing, discussing and defining of themes was an iterative process which involved discussion with all four researchers following a rights‐based approach. Five themes were agreed as an outcome of discussions in relation to the potential themes. Thematic analysis is an appropriate data analysis method allowing the researchers to reflect and self‐question their assumptions and expectations of their study while producing themes that tell a story of the results (Braun and Clarke 2022). A narrative summary ensures to relate to results with the review objective.

## 3 Results

### 3.1 Selection of Literature

**Figure 1:**
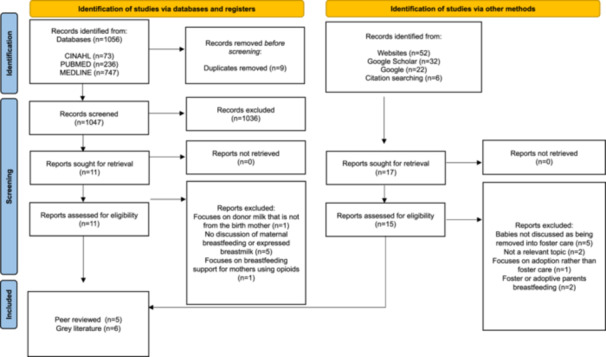
PRISM‐ScR.

In total, 1068 sources were identified through the search strategies. After the title, abstract and full‐text screening, 11 information sources met the inclusion criteria: five peer‐reviewed papers, and six grey literature materials, including two National Health Service (NHS) guidelines and policies (Figure 1).

**Figure 2:**
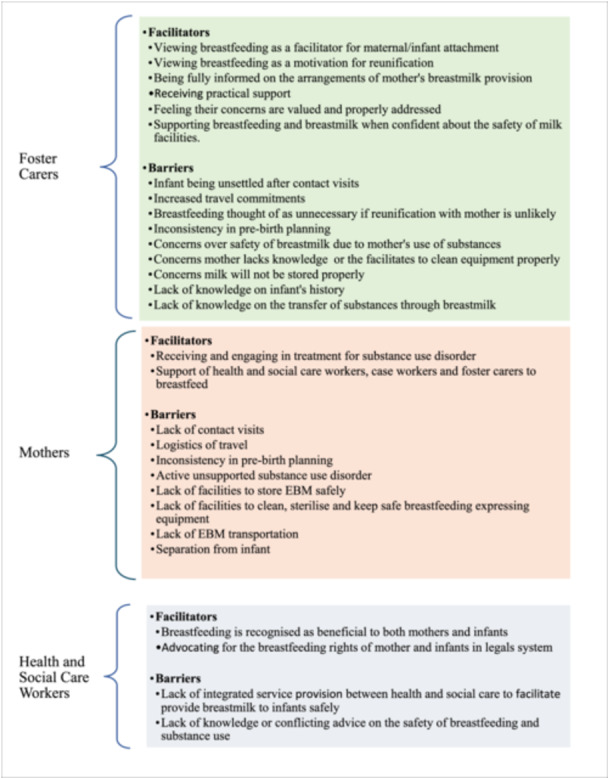
A summary of the facilitators and barriers to breastfeeding and EBM support for foster carers, mothers and health and social care workers.

The five peer‐reviewed papers were published between 2014 and 2022 (Blythe et al. 2022; Blythe et al. 2021; Gribble and Gallagher 2014; Gribble 2021; Paynter 2019). All were conducted in high‐income countries: three in Australia (Blythe et al. 2022; Blythe et al. 2021; Gribble 2021), one in Canada (Paynter 2019) and one in the UK (Gribble and Gallagher 2014). Two papers (Blythe et al. 2022; Blythe et al. 2021) report from the same original research study, one paper presents two case studies (Gribble and Gallagher 2014) and the final two papers are clinical opinion pieces (Paynter 2019; Gribble 2021). The full characteristics of the peer‐reviewed literature are presented in Table 3.

**Table 3:** Charting the data, peer‐reviewed literature.

Author and year	Country	Study aims	Participants and sample size	Research methodology	Method of feeding	Professionals involved	Study findings and/or issues addressed
Blythe et al. (2022)	Australia	To explore foster carers’ views on feeding infants in their care expressed maternal breastmilk and the facilitation for mothers breastfeeding during contact visits	Foster or kinship carers who had cared for at least one infant between 2013 and 2018. *n* = 184	Online survey containing	Foster carers' experience of infant‐feeding: EBM.	Social worker; case worker child protection worker	30% had cared for infants who were breastfed during contact visits. 28% answered yes to receiving EBM for an infant in their care Foster carers positive views: breastfeeding is a facilitator for attachment and reunification Foster carers negative views infants harder to settle after contact visits, increased travel; not beneficial if reunification was not possible; concerned to handle breastmilk
Blythe et al. (2021)	Australia	To explore the facilitation of breastfeeding in the out‐of‐home care and foster carers management of EBM	Foster carers who had experience caring for an infant (0–12 months old) within the last 5 years *n* = 184	A descriptive online survey	EBM and breastfeeding	Foster care agency	Foster carers concerned infant exposure to harmful substances through EBM Foster carers lacked information regarding safety of substance use and breastfeeding Identified the need for integrated care between the health and child protection systems to facilitate access to safe breastmilk
Gribble and Gallagher (2014)	UK	Comparison of two case studies of breastfeeding infants placed in foster care	Two infants and their mothers Baby E— birth mother was 18 years old Toddler A—birth mother was a respite carer for children with disabilities	Case studies	Baby E: Breastfeeding at time of removal; expressing milk for transport; breastfeeding in visit times Toddler A: Breastfeeding – mother and infant allowed to stay together at grandparents' house	Health visitor; local authority welfare concern; family care worker; child protection paediatrician at hospital; nursing matters charity; social workers	Recommendation for child protection authorities to support the breastfeeding rights of children through policies and training
Gribble (2021)	Australia	To share experiences of providing breastfeeding expert reports to assist Court decision‐making	Not applicable	Practice and policy article	Breastfeeding	Child protection services; criminal and family law; health professionals and breastfeeding specialists who can provide expert knowledge	Experts must only provide a report based on their area of expertise. They need to know the Court's Code of Conduct. Reports must present a balanced, non‐judgemental viewpoint to enable a judge to make an informed decision Include: Professional qualifications, expertise, current breastfeeding guidelines, relevant background of the child, the mother and their feeding history and a personalised evidence‐based analysis of the benefits of breastfeeding for the mother and child Address potential concerns such as maternal substance use and provide alternative arrangements for the mother and child that could support breastfeeding continuation Court reports can bring the issue of a child's right to breastfeeding and/or breastmilk before judges
Paynter (2019)	Canada	To provide clinicians with a description of the content to include in a clinical opinion letter supporting breastfeeding dyad to remain together	Not applicable	How‐to descriptive written piece	Breastfeeding	Nursing; family law; child protection services; clinicians	United Nations Convention on the Rights of the Child (UNCRC), Best Interest of the Child and the Right to Enjoy Health Support women to breastfeed when they enrol on an opioid agnostic programme Breastfeeding is recommended by WHO as it benefits both mother and infant Breastfeeding is only successful if facilitated by continuous contact

The publication dates of the six grey literature sources ranged from 2019 to 2023. These sources of information included two policies, one guideline, one opinion piece, one manual and one report. Again, all were from high‐income countries: three from Australia, one in Canada and two from the UK. The full characteristics of the grey literature are presented in Table 4.

**Table 4:** Charting the data, grey literature.

Author/website date/country	Information type	Title	Aims of the information source	Who is the resource aimed at?	Infant‐feeding discussions
Department of Children and Families (2019) Connecticut USA	Policy	Specialised Child Welfare Subject Matter: Breast Milk Feeding During Placement	To outline how to facilitate breastfeeding by the mother or through stored breast milk for infants in out‐of‐home placement	Health and social care professionals who are involved in the out‐of‐home placement of a breastfeeding infant, and the mother	Discussions should involve the social worker, parents and wider multidisciplinary Team (MDT) Reasons that breastfeeding might be discontinued include mothers' choice, active substance use and transportation logistics If breastfeeding cannot be continued at the time of removal, an alternative nutrition source should be available Foster teams should support foster families who are willing to facilitate the continuation of breastmilk feeding the infant
The Conversation Website Gribble and Smith (2019) Australia	Informed comment written by academic experts	Mums in Prison or Whose Infants Are in Care Need Breastfeeding Support too	To present some of the breastfeeding challenges faced by mothers who are involved with both the child protection and criminal justice systems	Those interested in the topic of breastfeeding or EBM for infants who are placed in foster care or whose mothers are incarcerated	Australia's new National Breastfeeding Strategy Breastfeeding should be protected through policy, and where separation occurs, mothers are given support to breastfeed during visits or EBM Recognises the logistical challenges of mothers and infants being separated and maternal drug use Supporting breastfeeding improves maternal health, reduces health inequalities, reduces reoffending, the cost of women in prisons and foster care Advocates that any mother who interacts with child protection systems should be supported to breastfeed
Department of Child Safety, Seniors and Disability Services Child Safety Practice Manual (2023) Section ‘Supporting the breastfeeding of a child in care’ Queensland Australia	Government procedures manual	Meet a Child's Health and Wellbeing Needs	To provide points to be discussed with parents and foster carers regarding their breastfeeding wishes	Health and social care professionals involved with parents who are having a child placed in out‐of‐home care and wish to continue facilitating breastfeeding or transportation of EBM	Physical and emotional benefits of breastfeeding. Breastfeeding should continue for infants in foster care and parents should be involved Healthcare Professionals (HCP) should be involved to discuss and minimise risks with safety of breastmilk Foster families should be provided with information regarding the breastfeeding arrangement, have their views and capacity to facilitate breastfeeding listened to, and be supported with practical arrangements
Ministry of Children and Family Department and Representative for Children and Youth (2018) British Columbia Canada	Joint Special Report	Promoting Access to Breastfeeding in Child Welfare Matters: A Joint Special Report	To present common challenges that affect best practice in service delivery to families where the Ministry of Child and Family Development (MCFD) or a Delegated Aboriginal Agency (DAA) were involved, regarding breastfeeding mothers	Submitted to the Legislative Assembly of British Columbia and available to the public	Social workers were faced with conflicting advice regarding the safety of breastmilk for mothers who were on a methadone management programme or using substances There was inconsistent pre‐birth planning for breastfeeding that involved the birth parents. Some cases involved parents being told that they would have access to breastfeed but encountered challenges for visits Service could not provide EBM transportation services
Northumbria Healthcare, NHS Foundation Trust (2020)	Guideline	Guideline for Offering Birth Mother's Expressed Breast Milk to Infant Living With Foster Parents	To provide a list of points for MDT to consider before expressed breastmilk being offered to a child in foster care or other alternative care	HCP and wider MDT involved in the facilitation of expressed breastmilk for infants living in foster care	Breastfeeding when the mother has contact or transportation of EBM Considerations on unstable drug use, neglect, fabricated or induced illness, hygiene concerns, education of mothers regarding EBM, storage and transport, equipment, involving infant‐feeding co‐ordinator, a personalised plan to support the mother in breastfeeding or EBM, and plan for feeding
National Health Service, Lothian (2021)	Policy	Expressed Breastmilk: Information for Carers of Vulnerable Infants	To provide an information pack for carers that outlines and supports their role and responsibilities regarding expressed breastmilk	Carers who have an infant in their care who is receiving expressed breastmilk	Most substances pass between mother and baby through breastmilk. However, the volume of substance passed to the baby is usually very small, less than 1% Health and social care practitioners should discuss with mothers their current substance use to decide if the benefits of breastfeeding outweigh the risks to the baby. Mothers with continued drug use, such as cocaine should be advised not to breastfeed Discussions around Neonatal Abstinence Syndrome (NAS) and the safe introduction of formula milk for mothers who discontinue breastfeeding Responding to cues rather than setting a time routine. Safe storage and transportation of EBM, correct temperature for storing and expiry dates

### 3.2 Thematic Analysis

The following five themes were developed: Is this safe; Substance use: Protecting the breastfeeding rights of mothers and infants; Making milk mobile through breastfeeding and EBM; Where are the policies; and Attitudes around breastfeeding.

#### 3.2.1 Theme 1 | Is This Safe?

Five papers findings contributed to this theme that discusses the safety concerns of breastmilk or EBM from foster carers and health and social care workers (Blythe et al. 2021; Blythe et al. 2022; Ministry of Children and Family Department and Representative for Children and Youth 2018; Northumbria Healthcare, NHS Foundation Trust 2020). There were concerns around harmful substances in the breastmilk, divergent information relating to breastmilk safety and concerns for foster carers in handling EBM.

Two papers found that foster carers were concerned that the infants in their care were being exposed to harmful substances via breastmilk (Blythe et al. 2021, Blythe et al. 2022). This was reported as being a barrier to the provision of breastmilk as foster carers were at times choosing to discard EBM (Blythe et al. 2021). Additional safety concerns included poor hygiene or sterilisation of expressed and transported milk (Blythe et al. 2022). The Paynter (2019) paper discussed supporting and advocating for safe breastfeeding when the mother is on an enroled methadone or buprenorphine programme.

The Canadian report (Ministry of Children and Family Department and Representative for Children and Youth 2018) discussed the challenges social workers experience navigating conflicting medical advice on the safety of breastmilk from mothers on methadone replacement therapies or actively using substances. In the UK, the Northumbria Healthcare, NHS Foundation Trust (2020) guideline highlights that HCPs must consider if mothers have access to appropriate facilities for the safe and hygienic expression of breastmilk, including clean equipment, suitable storage facilities and if the EBM be transported in a timely manner.

Blythe et al. (2022) found that foster carers also had concerns about their own exposure risk when handling EBM. This was echoed in the Blythe et al. (2021) paper, where foster carers' voiced concerns about the risk of being exposed to substances through breastmilk. They lacked knowledge and confidence in handling EBM as they did not know if the mother was using substances.

These findings suggest that there is a knowledge deficit regarding the transmission of substances through breastmilk for foster carers but also exists for health and social care workers. There is also evidence to suggest that foster carers' have a lack of information on the infants in their care, as well as on whether substance use is a concern regarding the mother.

#### 3.2.2 Theme 2 | Substance Use: Protecting the Breastfeeding Rights of Mothers and Infants

This theme explores the ways the multi‐disciplinary team can support breastfeeding among mothers with substance use disorder and who are at risk of infant removal (Paynter 2019; National Health Service, Lothian 2021; Department of Children and Families 2019; Northumbria Healthcare, NHS Foundation Trust 2020; Gribble 2021). Gribble (2021) advocates that when an infant is breastfed, a report should be written by someone with breastfeeding expertise in order that a child's right to breastfeeding is properly considered by judges in child protection proceedings.

Linking to the safety concerns of breastmilk, Paynter (2019) advocates for mothers to breastfeed who are enroled on prescribed medication use for opioid use disorder (MOUD) and suggests that other clinicians and healthcare professionals should too.

Improving collaboration between nursing, family law, and Child Protection Services may facilitate earlier introduction and communication of the major considerations regarding rights to breastfeed and value of breastfeeding and reduce the harm of infant‐parent separation.

(Paynter 2019, p.350)

Evidence suggests that even in high maternal doses, breastfed infants receive less than 0.1 mg/kg/day of methadone through their mother's milk (Bogen et al. 2011). Breastfeeding reduces the severity of Neonatal Abstinence Syndrome and improves the overall health of the mother and bonding with their infant (Dieterich et al. 2013).

Assessing the risks associated with breastfeeding and substance use is a shared responsibility between the mother, and health and social care professionals (National Health Service, Lothian 2021). Prescribed and non‐prescribed substances may be transmitted in human milk (National Health Service, Lothian 2021). However, depending on the type of substance, the amount can be negligible.

The Northumbria Healthcare, NHS Foundation Trust (2020) guideline advises that mothers adhering to prescribed agnostic therapies, who are not actively using other non‐prescribed substances should be encouraged to continue breastfeeding or expressing their milk. However, some substances cannot be safely used during breastfeeding. For example, if some non‐prescribed substances, for example, cocaine, are actively being used by mothers, breastfeeding should not be advised as this may cause harm to the baby (National Health Service, Lothian 2021; Department of Children and Families 2019). To ensure breastfeeding is safe in the context of substance use, HCPs should liaise with other professionals who are experts on this topic, to determine an infant‐feeding plan for babies entering foster care that takes into consideration the mother's current drug and alcohol use. Open and honest conversations between the mothers and their health and social care professionals can help to facilitate safe breastfeeding practices, which also encourages the mothers parenting and caring responsibilities.

These findings suggest that there is a lack of awareness from both foster carers and health and social care professionals on the rights to breastfeeding for mothers and their infants. Clinicians should appropriately promote, support and advocate for mothers to initiate and continue breastfeeding.

#### 3.2.3 Theme 3 | Making Milk Accessible Through Breastfeeding and EBM

Six papers discussed the transportation logistics and storage difficulties for facilitating breastfeeding and EBM (Blythe et al. 2022; Gribble and Gallagher 2014; National Health Service, Lothian 2021; Gribble 2021; Northumbria Healthcare, NHS Foundation Trust 2020; Department of Children and Health 2019; Ministry of Children and Family Department and Representative for Children and Youth 2018).

Related to breastfeeding, in the Blythe et al. (2022) paper, foster carers reported they did not always feel that the increased visitations required for infants to be breastfed were appropriate for the infant:

I believe it is not in the best interests of the child to be transported by different strangers every day at such a very young age. Disrupting feeds and sleep times seems to not be in the best interests of any child.

(Blythe et al. 2022, p.6)

This showed that foster cares think that transportation to facilitate breastmilk might not be in the interest of the infant. However, Gribble (2021) promotes that contact for breastfeeding mothers is preferably daily, or at least three times per week. This includes babies possibly requiring more than 4 h of contact two times per week if they are refusing EBM or if the mother is having difficulties in expressing her milk. Despite this, foster carers feel that increased visitations require them to be more flexible and increased travel times. Some foster carers felt that consideration must be given to other children in the foster home:

Unrealistic. A carer would be unable to constantly make the child available to be fed at a rate that be of any benefit to the child. It would also be a significant inconvenience to the foster family to the point where care of the other child would be compromised. I would decline an offer of a placement which involved feeding of this kind. (Blythe et al. 2021, p.6)

One mother in the Gribble and Gallagher (2014) case study had one contact visit per day on weekdays. The mother hired a breast pump and reported repeatedly contacting social workers to inform them that she had EBM that she wished to be transported to her infant. The mother's EBM was never transported and within 6 weeks of the infant's placement in foster care, the infant was refusing to be breastfed, and by 2 months breastfeeding had stopped. The lack of guidelines for the breastfeeding infants in foster care was criticised by the panel involved in the case:

The panel was also disappointed that guidance had not been drawn up for staff in respect of the management of breast‐fed babies that have to be received into care in any circumstances but particularly in emergency situations.

(Gribble and Gallagher 2014, p.8)

The National Health Service, Lothian (2021) provides detailed guidance for carers of vulnerable babies on the correct storage temperatures and expiry dates of EBM. The guidance states that only EBM that has been stored correctly should be transported between mother and foster carer. The guidance goes on to state that EBM must be stored in a sterilised container, in the body of a fridge at below 4° for up to 5 days; EBM should be stored in the ice compartment of a fridge for up to 2 weeks, and longer‐term storage should be in a freezer at –18° or below for a maximum of 6 months. EBM should be transported in a cool bag with ice packs, or in a cooler, and any equipment used for collecting or storing breastmilk should be exclusive to each mother and her baby. The guidance also discusses that carers should throw any EBM left over at the end of the feed. In additional to the National Health Service, Lothian (2021) guidance, The Northumbria Healthcare, NHS Foundation Trust (2020) policy discusses the hygiene concerns and risks associated with EBM include having the facilities for EBM to be transported in a timely manner. This includes mothers having a fridge for storing their milk and having a cool bag for it to be transported.

An additional policy from the Connecticut Department of Children and Families (2019) highlights that breastfeeding might be discontinued due to EBM not being transported to the infant. This was similarly discussed in the Canadian Report (Ministry of Children and Family Department and Representative for Children and Youth 2018), which emphasised challenges including mother's EBM not being transported to their infant.

It appears that foster carers require more support with the logistics and planning of contact visits. Mothers who wish to continue breastfeeding their infant in foster care may need to be provided with expressing, sterilising, storage and transportation equipment. Additional logistical solutions could include mothers travelling to their infant who is in foster care, rather than the infant travelling to the mother (Gribble and Gallagher 2014), which could help to eliminate some of the travelling difficulties raised by foster families.

#### 3.2.4 Theme 4 | Where Are the Polices?

Three papers contributed to this theme that discussed a lack of policies that protect the rights of mothers who are breastfeeding their infants who in foster care (Gribble and Gallagher 2014; Blythe et al. 2021; Gribble 2021). This explores recognition of international breastfeeding recommendations, increasing training and integrated care between health and social care workers.

An increased attention for breastfeeding in local policy was highlighted in both the Gribble and Gallagher (2014) and Blythe et al. (2021) papers. In addition, Gribble (2021) highlights that when expert opinion letters are being written for courts involved in the care proceedings on a breastfed infant being removed into care, they should be aware of the national and international recommendations for breastfeeding. This includes the UNCRC in relation to human rights in access to breastfeeding and human milk. Considering these points, Gribble and Gallagher (2014) highlight that greater attention on policies and training from child protection services could help to protect and support the rights of breastfeeding children in foster care.

It is recognised that there was a lack of focus on the rights of mothers (Gribble and Gallagher 2014). The European Court of Human Rights advocates for an increase in guidelines that focus on a mother's right to breastfeed when their child is removed. Gribble (2021) also discusses that supporting mothers to breastfeed in this context can support mothers who have had their infants removed in their caregiving capacity. In addition, mothers have a right to continue breastfeeding and breastfeeding charities criticise the lack of guidelines that focus on the care and support of the breastfeeding infant who is in care (Gribble and Gallagher 2014).

#### 3.2.5 Theme 5 | Attitudes Around Breastfeeding

Three papers contributed to this theme that explored foster carers' attitudes and experiences around breastmilk consumption for babies in foster care (Blythe et al. 2022; Blythe et al. 2021; Ministry of Children and Family Department and Representative for Children and Youth 2018).

Foster carers explored on the positive bonding effects of breastfeeding for the mother‐infant dyad. In the Blythe et al. (2022) paper, foster carers saw breastfeeding as a facilitator for mother and infant attachment, and they felt that breastfeeding or expressing milk provided the mothers with the motivation to work towards unification.

Breastfeeding could help the birth mother to bond with her baby, giver her positive hormones etc. Which could help her be a better mother and help get her baby back, soothing for mother and baby, breast milk is best for baby.

(Blythe et al. 2022, p.5).

In the Blythe et al. (2021) paper, foster carers were asked *‘*are you supportive of the infant having frequent contact with their mother to enable breastfeeding?’ Most foster carers (49%) stated they were unsure if they were supportive of frequent contact and less than 10% answered that they did not support, showing attitudes of hesitation in supporting contact to enable breastfeeding. Some foster carers felt that infants were harder to settle after visits and questioned if breastfeeding was the right infant‐feeding method if reunification was not possible (Blythe et al. 2022). Even with these challenges, 40% said they would be supportive showing that, based on a complex health and social care issue, with the correct policies, guidance and awareness, this percentage could be increased.

This theme revealed attitudes around breastfeeding. This is an important aspect to consider in the facilitation of any method of breastmilk consumption for infants in foster care. Further research is needed regarding understanding the perspectives of mothers.

## 4 Discussion

The aim of this scoping review was to explore what support is available for health and social care workers, foster families and mothers with infants in care in providing breastfeeding and EBM feeding support and to identify barriers and facilitators for breastfeeding and EBM in foster care. The results of our review show that although the numbers of children in foster care is significantly high in the UK and around the world, there is a startling lack of peer‐reviewed and grey literature addressing the issues associated with breastfeeding and EBM for this population. Our review found only 11 sources on this topic, from a worldwide perspective, with Gribble being coauthor of 5 of these papers (Blythe et al. 2021, 2022; Gribble and Gallagher 2014; Gribble and Smith 2019; Gribble 2021). This lack of evidence highlights the imbalances placed on the topic of breastfeeding and access to breastmilk as a public health priority for protecting the health and development of society's most vulnerable children and their mothers.

### 4.1 Research Gaps

One of the main findings of our scoping review suggested that there is a concern around the safety of breastmilk, particularly regarding substance use. Additional research has shown that the transfer of methadone in breastmilk is small and that when a mother is adhering to a methadone treatment programme, breastfeeding can prolong the illicit substance‐free period (Jambert‐Gray, Lucas, and Hall 2009; Demirci, Bogen, and Klionsky 2015). Both the American College of Obstetricians and Gynaecologists and the American Association of Paediatrics recommend continued breastfeeding for women who are adhering to programmes using prescribed MOUD, with evidence that breastfeeding is associated with a reduced likelihood of pharmacological interventions for neonatal opioid withdrawal, compared to infants who are formula‐fed (The American College of Obstetricians and Gynaecologists 2021; Sachs and Committee On 2013). Breastfeeding guidelines should outline evidence‐informed breastfeeding contraindications for health and social professionals (Busch 2016).

Whilst breastfeeding or EBM is safe under the care and support of expert healthcare professionals in the context of prescribed MOUD, professionals should have ongoing discussions with mothers about the potential risks associated with the use of illicit substances and excessive alcohol whilst breastfeeding (Harris et al. 2023). To support mothers who are using substances with infants in foster care, and who wish to continue breastfeeding, discussions should be held that involve the mothers, foster families, social workers and other health and social care professionals. We know from our review that a facilitator to breastfeeding for this group included foster families supporting mothers to breastfeed or express their milk if they are engaged with a substance use programme (Paynter 2019). However, there was a clear concern from some foster carers and social workers on the safety of breastmilk. In addition, there appears to be a particular research gap on how best to communicate evidence‐based information on the safety of breastmilk regarding substance use to foster families and social workers (Blythe et al. 2021; Gribble and Smith 2019; Paynter 2019; Department of Children and Families 2019; Ministry of Children and Family Department and Representative for Children and Youth 2018; Northumbria Healthcare, NHS Foundation Trust 2020; National Health Service, Lothian 2021). Future research could focus on qualitative methodologies that aim to understand the lived experiences of foster families who have breastfeeding infants in their care when the mother is prescribed MOUD. This research could help to identify required improvements in the current information given to foster families to help facilitate positive attitudes towards supporting mothers to breastfeed.

An additional concern included the safe handling, storage and transportation of EBM. Mothers should be supported by being provided with the correct equipment to express and store their breastmilk, including access to a breast pump, appropriate cleaning equipment, a fridge and a means of transportation of EBM facilitated (Sibson and Crawley 2021). It also includes mothers having access to information and education on storage, cleaning guidance and feeling confident in executing this (Scott et al. 2020). We need a greater understanding of the barriers and facilitators for mothers breastfeeding or expressing their milk for their infants in foster care, from the voices of the mothers themselves. Future research should emphasise the experiences and views of this group of mothers, with the aim to understand the accessibility of information available to them and their opinions on how services and support could best meet their breastfeeding support needs. In addition, quantitative methods could determine the breastfeeding duration of mothers with infants in foster care, and in turn, explore the health outcomes of these infants.

### 4.2 Policy and Practice Gaps

Protecting the health, development and rights of infants in foster care needs to be a policy and practice priority. Gribble and Gallagher (2014) discussed the role that child protection services and local authorities have in upholding the rights of breastfeeding mothers and their infants in relation to the UNCRC international recommendations for breastfeeding. This includes working in partnership with those in legal systems to ensure that the interventions put in place for infants in foster care support and advocate for breastfeeding mothers (Gribble and Gallagher 2014; Gribble 2021). There needs to be greater emphasis placed on policy that is rooted in this international breastfeeding evidence. For example, judges who are involved in care proceedings for breastfeeding mothers and infants should be routinely provided with expert opinion reports on breastfeeding, which could in turn, contribute to improve the health and wellbeing of these vulnerable children and influence practice and policy making.

Our review found there was significant concern across multiple studies about substances being passed between mother and infant through breastmilk from both foster families and social care workers. As evidence previously discussed suggests, continued breastfeeding for women who are adhering to programmes using prescribed MOUD is recommended, and it could be that with the implementation of coherent and evidence‐based policy, concerns could be largely overcome. By ingraining this type of policy in our health and social care partnerships, foster families and social workers can be supported and reassured by health professionals with expertise in the field of toxicology and breastmilk. It involves non‐judgemental care practices of mothers, foster families and health and social care workers, which can come together in holistic discussions about infants in their care on a case‐by‐case basis.

Understanding the high rates of trauma among pregnant women and new mothers is important in the context of breastfeeding (Seng, D'Andrea, and Ford 2014). This is particularly pertinent for mothers who have their infants placed into foster care and who experience many health inequalities. When supported with infant‐feeding, breastfeeding can positively impact mother's trauma‐induced symptoms (Uynas‐Moberg et al. 2020). Ensuring mothers have support and an enabling environment of breastfeeding will support their breastfeeding rights (Radzyminski and Callister 2016). Gribble (2024b) suggests that this be achieved through the joint support of, for example, health and social care services, child protection, the justice system, third‐sector organisations and the education system. All these avenues need to recognise their role in advocating for breastfeeding, as well as proximity, and skin‐to‐skin for vulnerable mothers and infants. To achieve this, policy and guidelines are required to outline breastfeeding rights and support required for mothers, with instructions on how to express their breastmilk, including availability of equipment for expressing their milk, equipment for milk storage and up‐to‐date, evidence‐based, accessible information on safe expression and storage (Gribble 2024b). For mothers and infants who are separated, these steps can contribute to an increased awareness of the importance of breastfeeding (Gribble 2024b).

Policy and practical guidance are required for all professionals working within this field to have the appropriate training and skills to be able to support mothers on their right to breastfeed and EBM safely (Murphy et al. 2022; Gavine et al. 2022; Gribble 2024b). For example, foster care agencies should ensure that foster parents receive training on the importance of breastfeeding on physical and emotional health for mothers and infants, along with the practicalities, which in turn, could improve sensitive caregiving and attachment for mothers and their infants (Gribble 2024b). Our review found only three international guidance documents for social workers to support breastfeeding for infants in foster care, and in addition, only two NHS guidelines, which highlighted the importance of urgent evidence‐based policy and guidelines for health and social care workers in this field in the UK and across the globe.

Actively involving mothers who are having their infants placed into foster care in the decision‐making process around infant feeding is central to ensuring a mother‐child rights approach (Gribble and Gallagher 2014; Paynter 2019; United Nations 2022). It involves creating a space in which mothers feel safe and supported in their parental responsibilities. Gribble (2024a) highlights the importance of breastfeeding on maternal attachment and sensitive caregiving. The paper debates that breastfeeding duration of 6 months or more positively contributes to secure mother and infant attachment and is particularly important for mothers who are at risk of providing inadequate care to their infants (Gribble 2024a). It is necessary to create a strong foundation of policies and guidelines, as discussed above, which focus on safe breastfeeding practices for infants in foster care. Our scoping review identified facilitators and barriers to breastfeeding or EBM feeding for mothers who have their infants placed in foster care. By addressing these complex issues, we hope to showcase the importance of supportive guidelines and training resources for health and social care workers and foster families working with infants in foster care to facilitate the practice of safe and person‐centred breastmilk feeding. It is through collaborative working between health and social care partnerships that it will hopefully be possible to increase the support for families with infants removed into care to optimise nutritional and emotional support when social circumstances can create multiple barriers to the offer of breastmilk (Mason et al. 2022). As an imperative, vulnerable infants such as the ones in foster care need to have their rights protected through policy and practice, and this includes their right to receive breastmilk.

To the best of our knowledge, this is the first review to focus on support for breastfeeding and breastmilk consumption in the context of infants in foster care. Our review has some limitations associated with the scoping review methodology. We did not complete a critical appraisal as this is not required in scoping reviews, however, this can impact upon the rigour of the studies included. In addition, the broad search strategy adopted within scoping reviews can impact the transferability and generalisability of the results of our scoping review.

## 5 Conclusion

Our review highlighted that mothers' and infants' rights to breastmilk feeding require higher importance when infants are placed into foster care, which acknowledges national and international breastfeeding recommendations. The importance of breastmilk is widely acknowledged in healthcare policies and guidelines, and greater attention is required for this vulnerable group of mothers and infants in society to reduce health inequalities. Further research is required that focuses on improving guidance for health and social care workers and foster families to improve and facilitate breastfeeding and EBM feeding for mothers and their infants who are in foster care.

## Author Contributions

V.M. and M.W. developed the reviews aims and objectives. V.M. and M.W. completed the database searching. V.M., M.W., S.S. and C.B.‐D. completed the screening and analysis. V.M., M.W., S.S. and C.B.‐D. wrote the paper.

## Conflicts of Interest

The authors declare no conflicts of interest.

## Supporting information

Supporting information.

Supporting information.

## Data Availability

The authors have nothing to report.
