# Supplementary material for: Supporting Breastmilk Feeding for Infants in Foster Care: A Scoping Review
Source: Matern Child Nutr. 2025 Feb 10;21(3):e13810. doi: 10.1111/mcn.13810 (PMC12150123; doi:10.1111/mcn.13810)
Supplement: Supplementary file 1 — Supporting information. [file MCN-21-e13810-s002.docx]

| Database | Search Query | Date of Search | Results |
| --- | --- | --- | --- |
| CINAHL | Breastfeeding *OR* breast-feeding *OR* breast feeding *OR* breastmilk *OR* breast-milk *OR* breast milk *OR* complementary feeding *OR* combi feeding *OR* combination feeding *OR* lactation *OR* lactating *OR* expressed breastmilk *OR* expressed breast milk *OR* expressed breast-milk *OR* EBM  **AND**  foster care *OR* foster care system *OR* fostering *OR* out-of-home care *OR* kindship care | March 2023 | 90 |
