# Supplementary material for: Supporting Breastmilk Feeding for Infants in Foster Care: A Scoping Review
Source: Matern Child Nutr. 2025 Feb 10;21(3):e13810. doi: 10.1111/mcn.13810 (PMC12150123; doi:10.1111/mcn.13810)
Supplement: Supplementary file 2 — Supporting information. [file MCN-21-e13810-s001.docx]

| Author and Year | Title | Reason for exclusion |
| --- | --- | --- |
| Akhter et al 2013 | Health and Nutritional Status of Young Foster Children Attending a Diarrhoea Treatment Hospital in Bangladesh | Wrong outcomes – breastfeeding support for children in foster care is not the focus of this research. |
| Asiodu et al 2021 | Breastfeeding in incarcerated settings in the United States: A National Survey of Frequency and Policies | Wrong outcomes – study explores the adherence to pregnancy and breastfeeding policies within prisons in United States. |
| Bennett et al 2020 | A Call for Medicaid Coverage of Pasteurized Donor Milk for Foster Children | Wrong population – explores state policies on an infant receiving foster mother’s breastmilk and donor human milk |
| Busch 2016 | Clinical Management of the Breast-Feeding Mother-Infant Dyad in Recovery from Opioid Dependence | Wrong population – focus is on providing breastfeeding support for women who are opioid dependent |
| Edwards 2017 | An exploration of maternal satisfaction with breastfeeding as a clinically relevant measure of breastfeeding success | Wrong outcomes – focus is on the need to improve maternal satisfaction in breastfeeding support, it recognises that mothers with infants in foster care may need additional support but does not discuss how this could be achieved |
| Gribble 2005 | Breastfeeding of a medically fragile foster child | Wrong population – focus is foster mothers breastfeeding their foster child not the provision of the birth mother’s breastmilk. |
| Homeschooling my Kinetic Kids 2019 | Feeding your foster baby | Wrong population - focus is foster mothers breastfeeding their foster child not the provision of the birth mother’s breastmilk. |
| Huang et al 2012 | The significance of breastfeeding to incarcerated pregnant women: an exploratory study | Wrong population – participants were pregnant women in prison, study discussed their view of breastfeeding and their feeding plans once the baby was born |
| Jambert-Gray et al 2009 | Methadone treated mothers: pregnancy and breastfeeding | Wrong participants – focus breastfeeding among mothers who have a substance dependency |
| Johnston 2014 | A foster carers’ training package for home treatment of neonatal abstinence syndrome: facilitating early discharge | Wrong population – focus is the care of infants in focus care with neonatal abstinence syndrome. Breastfeeding is mentioned as beneficial but challenging and how to support breastfeeding is e not discussed |
| Mannel et al 2020 | Milking the System: A Case Study of Donor Milk for a Child in Foster Care | Wrong population – reports a case study of an infant receiving foster mother’s breastmilk and donor human milk (linked to Bennett et al 2020) |
| Sarkar et al | Characteristics of young foster children in the urban slums of Bangladesh | Wrong outcomes– paper compares the breastfeeding and weening history of children in foster care to their peers who are not in foster care. |
